# Uncovering the history of recombination and population structure in western Canadian stripe rust populations through mating type alleles

**DOI:** 10.1186/s12915-023-01717-9

**Published:** 2023-10-25

**Authors:** Samuel Holden, Guus Bakkeren, John Hubensky, Ramandeep Bamrah, Mehrdad Abbasi, Dinah Qutob, Mei-Lan de Graaf, Sang Hu Kim, Hadley R. Kutcher, Brent D. McCallum, Harpinder S. Randhawa, Muhammad Iqbal, Keith Uloth, Rishi R. Burlakoti, Gurcharn S. Brar

**Affiliations:** 1 Faculty of Land and Food Systems, The University of British Columbia (UBC), Vancouver, BC Canada; 2 Agriculture and Agri-Food Canada (AAFC), Summerland Research and Development Center, Summerland, BC Canada; 3 Kent State University, Stark Campus, North Canton, OH USA; 4 Department of Plant Science/Crop Development Centre, University of Saskatchewan, Saskatoon, SK Canada; 5 Agriculture and Agri-Food Canada (AAFC), Brandon Research and Development Center, Brandon, MB Canada; 6 Agriculture and Agri-Food Canada (AAFC), Lethbridge Research and Development Center, Lethbridge, AB Canada; 7 Faculty of Agricultural, Life & Environmental Sciences, University of Alberta, Edmonton, AB Canada; 8 British Columbia Pest Monitoring Network, Dawson Creek, BC Canada; 9 Agriculture and Agri-Food Canada (AAFC), Agassiz Research and Development Center, Agassiz, BC Canada

**Keywords:** *Puccinia striifor*mis f. sp. *tritici*, Stripe rust, Plant-pathogen interactions, Fungal mating type, Field pathogenomics

## Abstract

**Background:**

The population structure of crop pathogens such as *Puccinia striiformis* f. sp. *tritici* (*Pst*), the cause of wheat stripe rust, is of interest to researchers looking to understand these pathogens on a molecular level as well as those with an applied focus such as disease epidemiology. Cereal rusts can reproduce sexually or asexually, and the emergence of novel lineages has the potential to cause serious epidemics such as the one caused by the ‘Warrior’ lineage in Europe. In a global context, *Pst* lineages in Canada were not well-characterized and the origin of foreign incursions was not known. Additionally, while some *Pst* mating type genes have been identified in published genomes, there has been no rigorous assessment of mating type diversity and distribution across the species.

**Results:**

We used a whole-genome/transcriptome sequencing approach for the Canadian *Pst* population to identify lineages in their global context and evidence tracing foreign incursions. More importantly: for the first time ever, we identified nine alleles of the homeodomain mating type locus in the worldwide *Pst* population and show that previously identified lineages exhibit a single pair of these alleles. Consistently with the literature, we find only two pheromone receptor mating type alleles. We show that the recent population shift from the ‘*PstS1*’ lineage to the ‘*PstS1-related*’ lineage is also associated with the introduction of a novel mating type allele (*Pst-b3-HD*) to the Canadian population. We also show evidence for high levels of mating type diversity in samples associated with the Himalayan center of diversity for *Pst*, including a single Canadian race previously identified as ‘*PstPr*’ (probable recombinant) which we identify as a foreign incursion, most closely related to isolates sampled from China circa 2015.

**Conclusions:**

These data describe a recent shift in the population of Canadian *Pst* field isolates and characterize homeodomain-locus mating type alleles in the global *Pst* population which can now be utilized in testing several research questions and hypotheses around sexuality and hybridization in rust fungi.

**Supplementary Information:**

The online version contains supplementary material available at 10.1186/s12915-023-01717-9.

## Background

*Puccinia striiformis* f. sp. *tritici*, the cause of stripe or yellow rust disease, is one of the five most important wheat pathogens in Canada and several epidemics of the disease impacted wheat production over the last two decades [1]. Efforts to understand virulence phenotypes are ongoing [2–5] while genetic population structure studies [6, 7] were limited in the Canadian landscape. Studying the genetic population structure of the pathogen in a global context is important because rust pathogen propagules can easily spread from one country to another with wind currents and even inter-continental spread in wheat rusts is reported [8]. Presence of foreign incursions of *P. striiformis* f. sp. *tritici* races in Canada has been speculated [7] but no study presented evidence of such incursions or information on origin of such incursions. While our research group studied the genetic population structure of Canadian *P. striiformis* f. sp. *tritici* populations, we utilized our generated and publicly available genomic resources to characterize mating type alleles in the global pathogen population. Mating type alleles in rust pathogens remain largely uncharacterized and no study has yet utilized mating type alleles in answering biological questions relating to sexuality or population biology.

Mating in basidiomycete fungi such as rusts, smuts, and agaricomycotina depends upon a variety of factors including the development of sexual macrostructures at a certain life cycle stage, environmental cues, chemical signalling between individuals, and genetic compatibility. From a genetic perspective, non-self-recognition to facilitate mating in rusts is controlled by two unlinked loci: P/R (sometimes called *a* and equivalent to the *B* locus in agaricomycotina) and HD (sometimes called *b* and equivalent to the *A* locus in agaricomycotina). The P/R locus encodes pheromone precursors (*mfa*) and receptors (*Pra*) which must be compatible in order for prospective mates to signal to one another and initiate syngamy. The HD locus encodes two homeodomain genes (*bW-HD1* and *bE-HD2*) which need to be of different allelic specificity in each mate in order for their protein products to form heterodimeric bW/bE homeodomain transcription factors which regulate cellular development during mating, and subsequent fungal life cycle stages including maintenance of the dikaryotic state and controlling pathogenicity in various smuts and rusts [9–13]. In some basidiomycota, the P/R and HD loci have become linked, leading to a bipolar rather than tetrapolar mating type [14]. In other cases, the alleles no longer discriminate against self-fertilization or are not required for mating, leading to a bipolar or even unipolar mating type [10]. So far, all characterized cereal rust fungi are tetrapolar [11, 15], including *P. striiformis*, but most species in the genus have not had their mating loci characterized and in the wider *Pucciniomycotina* there are multiple examples of bipolarity [11, 12].

The biochemical and genetic mechanisms of pheromone signalling at the P/R locus are complex and have been more extensively characterized in the Agaricomycotina and Ustilagomycotina [11, 16–18]*.* A number of models for the locus in Puccinomycotina exist, most recently reviewed in [12], but overall: two discrete cells must carry complementary pheromones and receptors in order to initiate syngamy. In three wheat rust pathogens, i.e. *P. triticina*,* P. graminis* f. sp. *tritici*, and *P. striiformis* f. sp *tritici*, three *Pra* receptor genes belonging to the STE3 family have been identified with *STE3.2-1* likely being a non-mating type receptor, and *STE3.2-2* and *STE3.2-3* the likely mating type pheromone receptors and hence nucleus / haplophase-specific [13]. Additional short (<200 bp) genes speculated to encode pheromone precursors (*mfa*) have been identified but their role is poorly characterized. In well-assembled genomes, the *mfa2* gene appears well-conserved and is located in proximity to STE3.2-2, whereas *mfa1* and *mfa3* are associated with STE3.2-3 and may not always both be present [15]. The three STE3 genes in different species consistently segregate when organized using phylogenetic methods, clustering with their orthologues and not their paralogues from the same species. Where sequence information from multiple isolates is available, additional STE3.2 alleles in rusts have not been identified, leading to the hypothesis that the three genes collectively comprise two complementary alleles, one of which is inherited with each haplotype, and one, STE3.2-1 is found in either haplotype. In this model, any dikaryotic rust cell will encode both *P/R* alleles, and haploid germ cells will have a 50% chance of being compatible with another germ cell.

The HD locus encodes a pair of homeodomain genes: *bW-HD1* and *bE-HD2* which are necessary for proper development of a viable, pathogenic fused dikaryon [11]. In *Pucciniales *spp., the genes are ~1800 and 1200 bp in length, with two and one introns respectively, encoding ~600 and ~400 amino acid length proteins [13]. Each protein exhibits three domains: at the N terminus, a Variable domain, a central structured Homeodomain, and a Constant domain at the C terminus. bW-HD1 and bE-HD2 are entirely dissimilar on an amino acid (AA) level, except for some (~50% AA similarity) in the homeodomain region. Evidence from heterologous systems indicates that the two gene products physically associate in the cell and act via DNA-binding activities of the homeodomain [18–20]. The mechanism for non-self-recognition is that variable domains of the same mating type prevent dimerization. Experiments with chimeric variable domains indicate that a relatively small alteration to the variable domain is enough to permit interaction [21–23]. Without the dimerized proteins, mating will not produce viable dikaryotic hyphae; the normal cellular growth process cannot proceed without this dimer active within the nucleus. In *P. triticina*, at least nine mating types have been identified (Guus Bakkeren, personal communication). Analysis of the published genome assemblies of *P. striiformis* f. sp. *tritici* identifies two alleles of each *Pst-bW-HD1* and *Pst-bE-HD2* in each genome on chromosome 4, although these are not always present in the published assemblies, and five distinct alleles of each *Pst-bW-HD1* and *Pst-bE-HD2* in total, with the P/R locus being on chromosome 9 and the related non-P/R gene STE3.2-1 present on chromosome 1 [15]. It has been hypothesized that maintenance of a plurality of mating types is evolutionarily unfavourable without selection pressure to maintain outcrossing in sexual reproduction [10, 24, 25]. Loss of mating type diversity through translocation to collapse HD and P/R into a single locus, as well as recombination events leading to self-fertility have both been observed in a number of related basidiomycete fungi [10, 11]; however, thus far the *Pucciniales* all exhibit maintenance of this system.

In cereal rusts, sexual recombination on the alternate host through pycniospores is known to occur regularly in *Puccinia coronata* [26], and *Puccinia graminis* [27] and has been shown to be possible in other species such as *Puccinia striifrormis*, and *Puccinia triticina* [28, 29]*.* Asexual reproduction, however, is the dominant mode of reproduction for studied *P. striiformis* populations, and most sampled isolates belong to genetically distinct clonal lineages. Rust fungi can also engage in somatic hybridization: a form of reproduction in which nuclei from multiple colonies are exchanged through somatic cells, giving rise to offspring with DNA from both parents [30]. The Ug99 race of *Puccinia graminis* was shown to be the product of somatic hybridization [31], as well as the 19NSW04 isolate of *Puccinia triticina* [32] and laboratory tests in many rust species have shown that the non-sexual progeny of co-cultivated admixtures on the primary host can exhibit segregation for traits which can only be explained by somatic hybridization. There is evidence to support multiple overlapping mechanistic pathways for genetic novelty here [30]. At one extreme, there is no exchange between haploid nuclei, and so entire haplotypes are inherited with no recombination or reassortment between chromosomes. This leaves a distinctive genomic signature which can be identified from modern chromosome-scale phased genomes, and was observed with the Ug99 *Puccinia graminis* lineage as well as the 19NSW04 isolate of *Puccinia triticina*. There is also evidence, however, of post-hybridization mitotic exchange in some cases of somatic hybridization, and even of pre-hybridization karyogamy as in the *Puccinia triticina* isolate Pt64 [33]. In this final case, there may be little to distinguish sexual and somatic hybridization after the fact. In this paper, we use somatic hybridization as an all-encompassing term for reproduction via the fusing of somatic cells from genetically distinct organisms, and parasexuality particularly to forms of somatic hybridization which exhibit some traits of sexual recombination such as crossing over or assortment of chromosomes from separate parent nuclei. This contrasts with heterokaryosis in which one nucleus is inherited intact from each parent. In all known cases, the progeny must carry complimentary mating type alleles as these are essential for normal cell cycle maintenance, and may regulate the interactions between nuclei during parasexual and heterokaryotic reproduction as well as sexual reproduction.

The concept of lineage in cereal rusts originates in the observation that in most studies the pathogen population is dominated by asexual descendants from a limited number of founder isolates, with genetic variation coming from mutations [34, 35]. *Pst* lineages are often formatted *PstS[n]*, although other descriptive systems exist. Not all samples have been or can be placed into a lineage. Without multiple samples of the same lineage to experiment with, there is no way to characterize the group, and not all individuals are successful in reproducing from year to year, so sometimes a novel isolate will be partially described and then disappear. Sometimes multiple scientists will describe the same lineage, and only realize it after comparable genomic data is shared between them.

Within this context, categorizing *P. striiformis* f. sp. *tritici* mating types worldwide might give clues as to the evolutionary history of particular *P. striiformis* f. sp. *tritici* lineages as a change in mating type away from expected within a lineage is an indication of non-asexual reproduction without the need for phased genome assembly and haplotype calling between related isolates. Using publicly available sequencing data from over 350 global *P. striiformis* f. sp. *tritici* samples, as well as from 35 Canadian isolates sequenced for this study, we identified nine distinct *P. striiformis* f. sp. *tritici* HD mating type alleles, and find that mating type combination can be easily assessed from whole genome/transcriptome sequence data, and corresponds 1:1 with genetic lineage for all of the five characterized *PstS* lineages assessed, as well as two clonal lineages from Europe and western Africa. In addition, we detect three population groups which exhibit multiple circulating mating types and do not show characteristics of being a clonal lineage descended from a single parent, in agreement with earlier work characterizing the global *Pst* population [7, 34, 36]. Finally, we connect a recent shift in the northern American *P. striiformis* f. sp. *tritici* population to the appearance of a novel mating type pair, suggesting that the *PstS1*-related lineage of *P. striiformis* f. sp. *tritici* is the product of a recent recombination event between *PstS1* and other existing lineages.

## Results

In order to characterize the history of recombination in the Canadian population of *P. striiformis* f. sp *tritici*, we first identified the set of alleles present at the HD locus across a global dataset. The global dataset consists of 332 previously published RNAseq and gDNA datasets including 17 Canadian samples, and 45 RNAseq datasets derived from samples taken from commercial crop fields in Canada and sequenced in this study. Mating type genes were identified and characterized using de novo assembled transcriptomic data derived from a variety of samples representing diverse lineages of *P. striiformis* f. sp. *tritici* (Additional File 1).

**Fig. 1:**
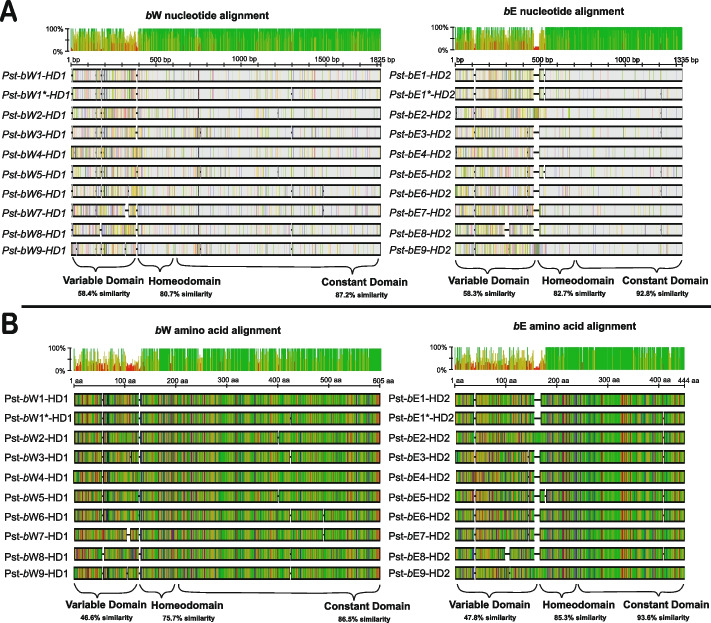
Nucleotide and amino acid diversity across 10 identified *Pst* HD locus alleles. **A** The CDS for each allele of *Pst-bW-HD1* and *Pst-bE-HD2* were aligned using MUSCLE and visualized in Geneious. Along the top of the alignments is a frequency plot indicating the percentage agreement with the most common nucleotide at that position. At the bottom of each alignment is the domain structure of the sequences in the alignment, and the average pairwise similarity within that domain across each allele. Each large rectangle represents the nucleotide sequence of a particular allele. Grey stretches indicate agreement with the most common nucleotide at that position. Coloured bars indicate a disagreement, shaded according to the base at that position (Red = A, Green = T, Yellow = G, Blue = C). Alignment gaps due to In/Del polymorphisms between alleles are represented as black horizontal bars, and a scale is provided above the sequences. **B** The amino acid alignments of each translated *Pst-bW-HD1* and *Pst-bE-HD2* allele were similarly aligned and cross-compared for agreement at each position. Along the top of the alignments is a frequency plot indicating the percentage agreement with the most common amino acid at that position. Each large rectangle represents the primary sequence encoded by that allele. Amino acids are coloured according to their polarity (Red = D,E; Green = C,N,Q,S,T,Y,U; Orange = A,F,G,I,L,M,P,V,W; Blue = H,K,R.) and gaps in the alignment are indicated by a black horizontal bar. A scale is provided above the sequences

A total of nine alleles for each of *Pst-bW-HD1* and *Pst-bE-HD2* were identified, encoding proteins of 593–602 amino acids, and 422–441 amino acids in length, respectively (Fig. 1, Additional File 2). All alleles encode seemingly functional proteins with the same predicted structure as other basidiomycete HD loci: an N-terminal Variable domain, a central Homeodomain, and a C-terminal Constant domain. The Homeodomain is the only domain with a predicted structure; the other domains are disordered. While the *Pst-bW-HD1* and *Pst-bE-HD2* coding sequences (CDS) have an average within-group pairwise nucleotide identity of 80.3 and 78%, respectively, their translated proteins are less well-conserved (77.7 and 75.2%). In all cases, the Variable domain has the lowest similarity between any two alleles, usually <60%. The *Pst-bW-HD1* and *Pst-bE-HD2* alleles do not share any meaningful identity outside of the Homeodomain. No indications of recombination between alleles were identified. For example, the *Pst*-*bW2-HD1* allele was always accompanied by a *Pst*-*bE2-HD2* allele, and the same for each other allele pair. *Pst-b1-HD* is unique in that a sub-variant, termed Pst-b1*-HD, was also identified, sometimes replacing only one of *Pst-bW1-HD1 or Pst-bE1-HD2* and sometimes replacing both. The sub-variant is identical in the variable region, but contains 12/36 and 17/41 SNP/amino acid polymorphisms in the other domains relative to *Pst*-*bW1-HD1* and *Pst*-*bE1-HD2,* respectively. We are unable to conclude if mating types *Pst-b1-HD* and Pst-b1*-HD are capable of mutual discrimination; however, it seems exceedingly unlikely given both experimental work in *Ustilago maydis* showing that the variable domain is the primary determinant of non-self-recognition and the lack of an exclusively *Pst-b1-HD* + Pst-b1*-HD mating type anywhere within the dataset [22].

Having identified the alleles present across this population, we then assessed all available nucleotide datasets for allele presence/absence by searching each dataset for *k*-mers contained within each allele. Nearly all samples (*N*=345/386) exhibited *k*-mer signatures for exactly two HD alleles. Datasets containing signatures of more than two alleles (*N*=18/386) are believed to be admixtures of more than one isolate or the result of sequencing error, while samples with fewer alleles (*N*=23/386) could represent as-yet uncharacterized alleles or simply low sequencing depth of one allele in the dataset (or both).

We also applied this approach to the P/R locus. The STE3 (*Pra*) family of hormone receptor encoding genes and the *mfa* family of hormone precursors have been characterized as determining *a-locus* specificity. Previous work identified three STE3 family genes in rusts: *STE3.2-1*, *STE3.2-2*, and *STE3.2-3*. *STE3.2-2* and *STE3.2-3* are most closely related to one another and are hypothesized to be biallelic receptor components of this mating locus, which each complement an *mfa*-derived hormone. *STE3.2-1* is thought to exhibit minor SNPs across isolates but not to the degree of presenting as distinct genes, as well as being present on a separate chromosome. We identified all three *STE3.2* genes in the Pst-130 datasets [37, 38]; however, *STE3.2-1* did not appear in any available transcriptome data, and we conclude that it is not expressed, or not expressed at a high level in either infected leaves or non-germinated urediniospores. *STE3.2-2* and *STE3.2-3* both appeared in the nucleotide data from nearly all isolates (Additional File 3: Figure S1). *STE3.2-1* was detected in genomic data from all isolates in which gDNA sequencing was performed. All *P. striiformis* f. sp. *tritici* samples in this analysis, therefore, share the same biallelic mating types at the P/R-locus. Isolates where *STE3.2-2* and *STE3.2-3* could not be identified did not contain any other observable STE3 sequence, or any HD sequence, indicating that these samples are likely to represent incompletely sequenced isolates rather than novel mating type specificities. Interestingly, while *STE3.2-2* and *STE3.2-3* are only 50% similar at the nucleotide level, and both are present in the raw reads of all genomic samples, BLASTn search of older assembled genomes were only able to identify at most one of these two genes intact, in addition to *STE3.2-1*. The most up to date phased genome (Pst 134E) successfully assembled all three *STE3* genes. In no cases were any *STE3* genes identified on the same genomic contig, unlike HD genes which are always found as a pair in head-to-head orientation. Collectively, these results support the model that the *STE3.2-2* and *STE3.2-3* genes are a biallelic pair and that their genomic loci may be collapsed by some assemblers.

**Fig. 2:**
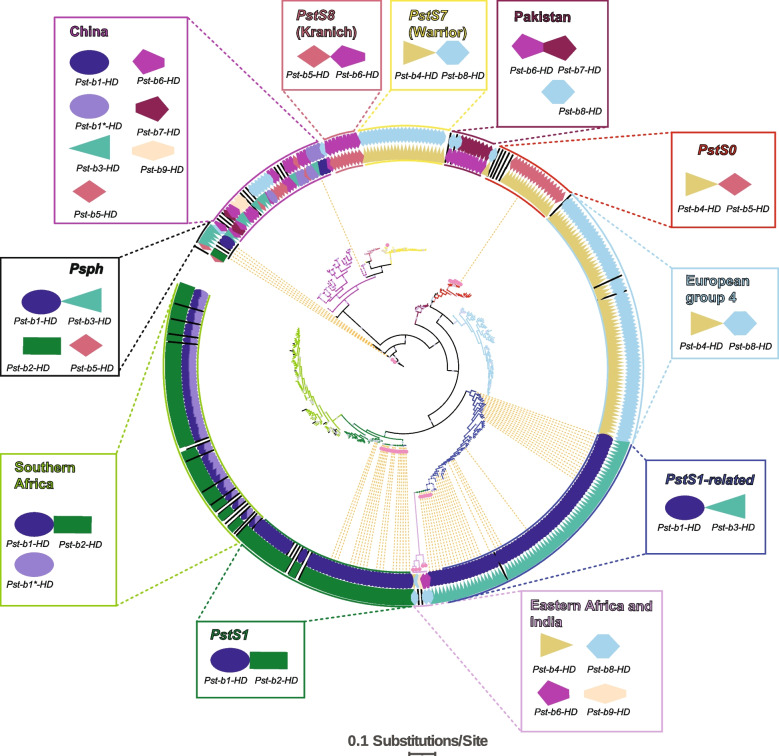
Maximum-likelihood phylogenetic tree detailing the relationship between *Puccinia striiformis* f. sp. *tritici* isolates, annotated with their mating type alleles. Dendrogram is derived from 206,376 SNPs across all datasets not excluded for poor coverage (*N*=370/386). Mating type alleles present in the isolate are shown as a pair of coloured shapes in the ring surrounding the tree. Unknown mating types are represented with a black bar. Clades are derived from bootstrap and STRUCTURE analysis and are displayed as coloured branches, matching outer ring colours, and matching text labels. Clade labels are supplemented below with the mating types identified within the clade. Samples from southern Africa exhibit a single gene with the *Pst-b1-HD* mating type and another with the Pst-b1*-HD mating type and so are shown with split colours. Bootstrap support >80% is shown with a small blue dot at the node. gDNA samples are highlighted with a pink dot just after the branch tip and can be observed to form a tight cluster within their clade. The tree was generated using RAxML and visualized/annotated in IToL and Adobe Illustrator

**Fig. 3:**
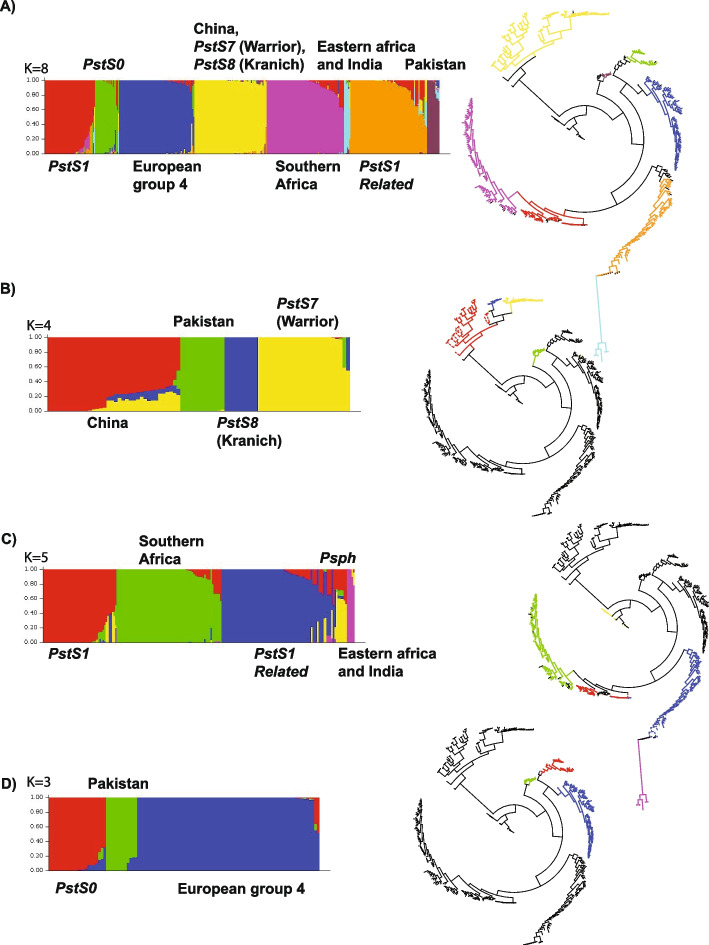
STRUCTURE analyses of the global *Pst* population identified only higher-order clades. STRUCTURE analysis of regional *Pst* populations identifies lower-order clades. Of the 12 population groups identified by previous work, only seven are identified in a global STRUCTURE analysis, with *K*>8 not identifying any additional groups. As other groups have reported, sub-analysis of major groups in STRUCTURE was able to correctly identify minor groups. Displayed are the primary, and secondary STRUCTURE analyses, sorted by proposed group (q) and the phylogenetic tree from Fig. 2 with the relevant clades highlighted for: **A** All samples. **B** Samples from China, Pakistan, *PstS7*, *PstS8* (India hidden for clarity).** C** Samples from groups *PstS1*, Southern Africa*, PstS1-related*, Eastern Africa and India, and *Psph. D* Samples from groups *PstS0*, Pakistan, and European group 4 (India hidden for clarity)

Having assessed mating types across the global *P. striiformis* f. sp. *tritici* population, we incorporated this data into a more conventional phylogenomic approach to assessing *P. striiformis* f. sp. *tritici* population structure. In brief, sequence data (RNA and gDNA) was aligned to the reference Pst-130.v2 genome [37, 38], and assessed for intragenic SNPs which were used to construct a maximum-likelihood tree that could be supplemented with HD allele data (Fig. 2, Additional file 4). This analysis was reinforced by using STRUCTURE [39] to identify likely genetic groups from the same intragenic SNP data, and cross-referencing these groups with the clades apparent on the tree. Similar to the work of Radhakrishnan et al. [36, 40], when using a global dataset, STRUCTURE was unable to resolve the more fine-grain distinctions between some closely related sub-clades apparent on the tree. However, taking the first-order clades and repeating the analysis successfully replicated the genetic groups apparent from the phylogeny (Fig. 3). Groups in our global phylogeny of *P. striiformis* f. sp. *tritici* form into two categories: clades descended from a single founder isolate, which represent a single, characterized clonal population such as *PstS7/Warrior* or *PstS0*, and population groups which contain a number of related isolates showing signs of admixture and which cannot be said to descend from a single isolate such as isolates sampled in China and Eastern Africa and India. Where a group neatly bounds around a previously described clonal lineage, we have annotated the group with that lineage, and where it does not, we have described the origin of the samples within the group. Of note is clade *PstS0*, which describes an extremely old lineage of circulating rusts and so while it can be considered a single clonal population, the individuals within that population exhibit far more intra-clade diversity than, for example, *PstS7/Warrior* which emerged in 2011. European group 4 refers to a group identified in [36] which we do not believe has a *PstS* designation. In that same paper, *PstS7* corresponds to European group 1, and *PstS8* to European group 5-1. In addition to short branch lengths and an unbalanced node structure indicating an asexual/clonal population structure in these clonal lineages, each exhibit only a single pair of mating type alleles throughout. Interestingly, European Warrior (*PstS7*) samples and older European samples, despite forming two robust and entirely distinct phylogenetic clades, each exhibit identical *Pst-b2-HD* + *Pst-b9-HD* mating type alleles, while Kranich (*PstS8*) samples which emerged contemporaneously to Warrior and are most closely related to Warrior exhibit *Pst-b5-HD* + *Pst-b6-HD* mating type alleles.

Three groups were not defined by a single clonal lineage, but rather collected from geographic regions of high diversity and clustering together, specifically those originating in China, eastern Africa and India, and Pakistan exhibit more mating type diversity with at least seven different HD alleles present in these clades in addition to longer within-group branch lengths and more evenly balanced nodes, indicating that recombination between isolates may be common in those regions. Relative to the large sample size of mostly clonal European and North American populations, it is likely that a substantial portion of *P. striiformis* f. sp. *tritici* diversity in these regions exists awaiting analyses, especially as these sample groups each only cover a short span of time (for example Chinese isolates were collected from 2015 to 2018). Two samples (W056 and USA 15.0349) in the ‘China’ group were not sampled within China and appear to represent isolates which spread into North America but were not successful enough to become established long term.

An additional finding of our work is that samples tended to cluster together based on their sequencing manner, i.e. gDNA samples within the *PstS1* clade cluster together relative to RNAseq samples from that same group indicating an unresolved systemic bias in tree construction (highlighted in Fig. 2, and detailed in Additional file 1). A tree constructed using RNAseq samples alone (Additional file 5) has the same clade topology as Fig. 2. The two trees have an edge similarity of 77%, going to 89% when only nodes with 80% or higher bootstrap support are considered: indicating that the overall clade assignment is sound, but that within-group comparisons of RNA and gDNA samples should be undertaken cautiously. We were not able to conclusively resolve the reasons for this discrepancy, but DNA samples exhibited a higher rate of heterozygous SNPs (88% s.d 1.9% ) than RNA samples (80% s.d 1.7%), possibly due to nucleus specific expression in mRNA giving the illusion of more homozygous SNPs and leading to longer branch lengths for RNA samples.

Having established the distribution of HD locus mating types across our datasets, and having placed them in the context of the global *P. striiformis* f. sp. *tritici* population, we investigated the North American *P. striiformis* f. sp. *tritici* population specifically. Up until now, analyses of North American *P. striiformis* f. sp. *tritici* lineages have grouped Canada and the USA into a single locale, reflecting their large land border and similar climates along each side of the border and due to the fact that stripe rust inoculum in Canada arrives from the USA via the ‘Puccinia pathway’ or wind trajectories along Pacific Northwest [1]. The very first incursion of *P. striiformis* f. sp. *tritici* to North America was in the early 1900s from Western Europe and so-called old races belong to the *PstS0* lineage which was restricted to regions west of the Rocky Mountains or southern Alberta. A very small number of isolates from North America were grouped in the *PstS0* clade (Fig. 2) indicating the lineage is present in North America, which is expected. The other predominant lineage in North America is *PstS1* and several isolates from the USA, Canada, Ethiopia, and Kenya were found to be part of this lineage. Prior to 2000, stripe rust was not a disease of concern to growers in Canada (except for southern Alberta); however, epidemics in 2000 and 2001 fueled by incursions (from eastern Africa) of races of the *PstS1* lineage to the USA and then to western Canada (via the ‘Puccinia pathway’ along the Great Plains) led to the disease becoming endemic by 2000–2001 [5, 7]. For the last decade, the majority of northern American *P. striiformis* f. sp. *tritici* isolates were believed to be *PstS1* lineage, with *PstS0* lineage races failing to outcompete this lineage [34, 36]. However, contrary to this belief and published literature, the majority of the North American races/isolates after 2015 belong to the distinct *PstS1-related* lineage and not *PstS1. PstS1-related* isolates were only detected in the USA and Canada unlike other lineages which are also present outside North America (Fig. 2). Other than *PstS0*, *PstS1*, and *PstS1-related* lineages, a single Canadian isolate W056/T210 grouped phylogenetically with the Chinese group of isolates, which is otherwise most closely related to the *PstS7/S8* (Warrior/Kranich) lineages. In our previous study [7], W056/T210 was named ‘*PstPr*’ lineage (Pr: probable recombinant) and it was proposed that the lineage is a foreign incursion (due to high telia production ability and close genetic relatedness to *PstS7/S8* isolates). The *PstPr* lineage was not successful at establishing itself in North America however [7], and no further samples with this unusual configuration have been detected. In the present study, we find that this race/isolate is genetically closest to isolates which were circulating within China in 2015, and share a single mating type allele. Given the lack of relatives sharing both mating types / haplotypes and its high telia production [7], it is possible that W056 was the result of a recent recombination event between a related Chinese lineage and North American lineage.

The HD locus mating type pair in *PstS1* is *Pst-b1-HD* + *Pst-b2-HD*, but in *PstS1-related* samples the mating types are *Pst-b1-HD* + *Pst-b3-HD*. It seems likely that the *PstS1-related* clade is the result of fusion between a *PstS1* individual and an individual from another clade with the *Pst-b3-HD* mating type; introducing genetic novelty into the North American population and founding a closely related sister clade to *PstS1*. As of yet, we have insufficient data to evaluate whether this fusion was sexual, parasexual, or heterokaryotic by interrogating for synteny between the chromosomes of *PstS1-related* individuals and a *PstS1* individual. The older *PstS1* clade is in fact more closely related to samples taken in the south of Africa than to the *PstS1-related* clade, as evidenced by their positioning in Fig. 2, their shared mating types, and STRUCTURE analyses which indicate that while they are distinct populations, they share substantial genetic overlap (Figs. 2 and 3). Indeed, when American and southern African samples are compared directly in STRUCTURE, *K*=5 identifies the *PstS1, PstS1-related*, southern African, and eastern African and Indian groups as separate clades, while a *K*>5 continues to describe these same clades but identifies *PstS1* and southern African samples as having a substantially shared genetic background (Additional File 3: Figure S2). These southern African samples date from 2014 to 2020, and as well as forming their own well-supported clade, and STRUCTURE group, share the common feature of at least one Pst-b1*-HD allele. As mentioned previously, this is the only example we found of a *Pst-b-HD* allele with substantial shared nucleotide and amino acid identity to another in the variable domain, and it is likely that one is a recently diverged variant of the other which has become widespread simply by being carried by the dominant asexual lineage in this region. Two interesting implications of this result are that the spread of this recent variant can be tracked by this single genetic marker alone and that these loci are not exclusively experiencing purifying selection pressure despite the lack of variation identified in other alleles.

Other than lineages detected in the North American *P. striiformis* f. sp. *tritici* population, our phylogenetic analyses supported a very diverse group of isolates originating mainly in China which the Warrior (*PstS7*) and Kranich (*PstS8*) lineages are derived from, a similar group originating in Pakistan, an unrelated group with samples from India and Eritrea (eastern Africa) including *Pst Race K and Pst Race 21*, and a western European lineage: Group 4 which appears to be a sister to the *PstS0* lineage in a similar manner to *PstS1* and *PstS1-related*, with each group sharing a single *Pst-b-HD* allele (*Pst-b3-HD*). The final clade, forming an outgroup on the tree is the *Psph* group (*Puccinia striiformis* f. sp. *pseudo-hordei*) which consists of samples collected from foxtail barley grass (*Hordeum jubatum*) as well as the reference genome for *Puccinia striiformis* f.*sp hordei* 93TX-2.

Bayesian STRUCTURE analyses supported the results of our phylogenetic analyses and identified 8 major groups within the global *P. striiformis* f. sp. *tritici* population (Fig. 3A, Additional File 3: Figure S2) as well as two minor groups (Fig. 3B). Our STRUCTURE analysis also supported the previously unresolved distinction between *PstS1*, *PstS1-related* and South African samples (Fig. 3C). *PstS0* and European group 4 were also separated by STRUCTURE analyses (Fig. 3D). It is important to note that *Psph* in the STRUCTURE analyses appears an admixture of multiple lineages from wheat (Fig. 3C) which is not surprising because the host for *Psph* can harbour rusts from both wheat and barley and participates in rust evolution differently from wheat [7].

## Discussion

Unlike previously published field pathogenomics studies on *P. striiformis* f. sp. *tritici*, our study placed North American isolates into two distinct lineages with a very clear distinction between *PstS1* and *PstS1-related*, which could be attributed both to a greater number of samples in each clade as well as the fact that we did not rely on a specific subset of genes [36, 41, 42]. Our approach was to use variation in all coding regions for maximum-likelihood phylogenetic analyses, the results of which were further supported by independent Bayesian analysis with STRUCTURE. This also suggests that the 242 genes described in Radhakrishnan et al. [36] might not be enough to capture global diversity in the pathogen populations as there are some indications of the division of North American population into two groups in that paper, but they were not able to conclusively separate the groups into two. A limitation of our approach, however, is the combination of gDNA and RNAseq-derived datasets which leads to an observable within-clade segregation between these two origins in the *PstS1* and *PstS1-related* clades, likely due to a combination of systemic error deriving from gDNA and RNAseq reads mapping differently to coding regions as well as differential expression between the two nuclei (i.e. genomic data may capture heterozygous SNPs which are missed in RNAseq data due to a lack of expression). Future -omics studies in *Pst* and other cereal rusts will hopefully take this factor into account, as we believe it is not always inappropriate to cross-compare these two sample types, but users must be aware of the limitations. Furthermore, it bears reminding that RNAseq data describes the condition of the sequenced colony and not the underlying genotype, and experiments which mis-estimate the contribution of both nuclei may not yield helpful results.

We identify a shift in the Canadian *P. striiformis* f. sp. *tritici* population after the year 2015 as the predominant lineage changes from *PstS1* to *PstS1-related*. The widespread prevalence of *PstS1-related* isolates over *PstS1* could most likely be attributed to increased fitness in the North American climate, increased urediniospore production or higher aggressiveness on Canadian wheat, but as yet we have no strong evidence for any particular hypothesis. Indeed, in our previous study [7], *PstS1-related* was hypothesized to be a recombinant lineage with higher telia production ability than the clonal *PstS1* lineage but the absence of an alternate host in North America should not lead this to favour the *PstS1-related* lineage over *PstS1*. The consequences of *PstS1-related* slowly replacing *PstS1* on Canadian wheat production have not been quantified, but we have not observed a major epidemic due to this incursion, only a single regional epidemic [5] and *PstS1-related* isolates do not appear to represent more aggressive or virulent races [data not shown].

This is the first study on the wheat stripe rust pathogen *P. striiformis* f. sp. *tritici* to identify and utilize mating type alleles in pathogenomics and population biology research. Identification of conserved mating type allele pairs across the majority of global lineages further supports the fact that the global population is largely clonal [34, 35, 43]. A larger diversity of mating type alleles was detected in China which is not surprising as the Himalayan region is the centre of origin and diversity of the pathogen [43] and several susceptible barberry (*Berberis* spp.) species as the alternate sexual host of the fungus have been identified from the region where sexual recombination is common [43–46]. From a small number of samples collected from Pakistan and India, there was considerable variation in mating type alleles and the presence of four alleles in each group suggested some level of sexual recombination which was also reported for isolates from Pakistan in another study [47]. However, the majority of the global lineages originates from a single founder race/isolate and do not show signs of recombination. If recombination is common then replacement of progenitor lineages is uncommon and the emergence of the *PstS1-related* lineage and replacement of *PstS1* as the dominant North American lineage seems to be an unusual event. Continued monitoring of global rust populations taking haplotype and mating type into account will help to resolve the question of whether hybridization is common and perhaps identify novel lineages as they occur in real time. Separately to the *PstS1* and *PstS1-related* split, we also identify the dominant southern African lineage of *Pst* as belonging to a unique lineage which probably diverged from *PstS1* some time before 2014, and can be tracked via its unique Pst-b1*-HD mating allele. More extensive sample characterization from these regions may even permit the rate of mutation in this allele to be measured as a proxy for its divergence from the founder isolate. These results also highlight the need for increased monitoring / study of the *Pst* population in countries bordering the Himalayas, as the relative number of samples from these regions of highest diversity is extremely small and often only covers a few points in time compared with the largely identical samples collected in European and North American agricultural locations. Further consideration should also be given to the physical size and geographical diversity of these countries as it is plausible that different regions of, for example, India have vastly different circulating rust populations.

It is clear that while mating types are a critical component of the *P. striiformis* f. sp. *tritici* genome structure and can complement other forms of analyses such as whole genome sequencing, RNAseq and of course phenotyping, they do not fully capture the diversity within a given sample as is made clear by the relationships between the *PstS1* and *PstS1-related* groups, as well as the totally shared alleles of the ‘Warrior’ and European group 4 lineages. We developed a method for detecting *b-HD* mating type alleles which can be included in other protocols for lineage identification or prediction because the majority of clonal lineages, such as those dominant in Europe, have unique allele combinations. Identifying a novel or unexpected mating type in a sample is a useful signifier that an unusual haplotype is present which only requires sequencing of a single gene. However, it must be remembered that most *Pst* lineages worldwide are uncharacterize and that *b-HD* allele cannot be used to properly characterize lineages which have not already been characterized or which carry alleles matching an existing lineage, only to identify the appearance of a novel strain and to complement other genomic markers.

Studying reproduction and mating types has always been important to understanding the evolutionary history and population dynamics of rusts, and modern advances in sequencing technology have made it significantly cheaper and easier to directly study the composition of the genes involved. Contemporaneously with this paper, work by Luo et al. [15] has assembled the *b-HD* and P/R loci of four cereal rust (*Puccinia*) species using a combination of new and public data, including five distinct alleles from three genomes of stripe rust (alleles *Pst-b1-HD, Pst-b2-HD*, *Pst-b4-HD*,* Pst-b5-HD*, and *Pst-b6-HD* in our study), and clarified the genomic relationship between the three members of the STE3.2 gene family. Additionally, recent genome assemblies of *Puccinia triticina* [33], *Puccinia graminis* [31], and the myrtle rust *Austropuccinia psidii* [48] have all paid special attention to these loci in quantifying the quality of their assemblies. This work identifying the global diversity of *b-HD* alleles in *Pst* permits investigation of the hypothesis raised in our previous study [7]: that the *PstS1-related* lineage is a somatic hybrid of *PstS1* and another Canadian lineage, as we predict the lineage is likely a somatic hybrid between *PstS1* and *PstPr* due to their shared alleles in *PstS1-related.* To collect further evidence for this claim, our research group is generating phased genome assemblies of all four lineages from Canada, coupled with chromosome confirmation, and virulence phenotyping data, which we intend to investigate for evidence of recombination which could support a heterokaryotic, parasexual, or sexual explanation for this lineage’s emergence.

## Conclusions

We show that the sequenced northern American *Pst* population has included individuals from at least four different worldwide population groups, but is overwhelmingly from lineages *PstS1* and *Pst1-related*. Furthermore since 2015, all sequenced samples originating from western Canada and most from the northern United States belong to the *PstS1-related* lineage. Using a combination of SNP-based maximum-likelihood tree and Bayesian STRUCTURE analysis, as well as a novel approach of mating type allele assessment, we show that *PstS1-related* is a close relative of *PstS1* but that it is not appropriate to consider them a single lineage as they share only a single haplotype. The distribution of genetic lineages of asexually reproducing pathogens such as *Pst* is of great concern to scientists, breeders, and growers as the emergence or incursion of novel lineages can lead to epidemics, while the existing lineages provide context for the deployment of cultivars with appropriate levels of resistance.

For the first time ever, we evaluate the global distribution of homeodomain-binding mating type gene alleles in *Pst*. We identify 9 mating type alleles in the available sequence data of *Pst* isolates around the world, and show they are both a simple proxy for lineage and can help to answer questions about the origins and relationships between different *Pst* lineages. While evidence for actively occurring sexual recombination in *Pst* is limited, recent papers have demonstrated that reproduction through somatic hybridization can occur in *Pucciniomcyotina*; leading to the emergence of novel lineages such as the virulent Ug99 lineage of stem rust [31]. As mating type genes regulate normal colony growth, we would expect both sexual and somatic hybridization to be mating type dependent. Finally, we find mating type diversity is highest in isolates collected near to the natural origin of stripe rusts, i.e. in countries bordering on the Himalayas.

## Methods

### Sample collection

In addition to 13 previously published gDNA samples which were included [7], we collected and sequenced the RNA of 43 Canadian rust sample datasets in this study. With the exception of W088 (collected in 1990), all Canadian isolates were collected between years 2005 and 2021. Additionally, one sample, AR00-05, was collected in 2005 from Arkansas, USA, by Dr. Eugene Milus (retired, U. of Arkansas, USA). Samples were collected as leaf tissue infected with a single lesion (single isolated stripe on the leaf) isolates and stored in RNAlater. Such samples are expected to be genetically pure as each successful stripe rust colonization event produces a single stripe along the vascular tissue of the leaf. Isolates described as SP (Single Pustule) have been passed through at least one round of purification through inoculation and spore recovery from a single pustule.

### DNA/RNA extraction and sequencing

Fifty-seven samples were sampled from Canadian fields between 2005 and 2021. Twenty-three were previously used for DNA extraction and WGS [7], and the remaining 34, along with AR00-05 isolate from the USA were used for RNA extraction and sequencing. Eighteen samples were not purified, and RNA was extracted directly from single lesion infected leaf tissue and sequenced with paired-end Illumina technology to a depth of ~10 Gbp. RNA extraction was performed following protocols described in Radhakrishnan et al. [36]. Seventeen samples were purified to single pustule isolates and RNA was extracted from urediniospores and sequenced with paired-end Illumina technology to a depth of ~5Gbp.

### Mating type gene identification and characterization

In order to characterize the history of recombination in Canadian *P. striiformis* f. sp. *tritici* populations, we first identified the set of alleles present at the HD locus across the global dataset [36–38, 40–42, 48–72]. In the Pst-130.v2 reference genome [37, 38], *Pst-bW-HD1* is represented by FUN_008986+FUN_008987 (partial annotations of a single gene) and by FUN_010468. *Pst-bE-HD2* is represented by FUN_008988 and FUN_010469. Using representatives from each identified clade, we performed de novo RNAseq-based transcriptome assembly using the Trinity [73] software package with default parameters. As the *Pst-bW-HD1* and *Pst-bE-HD2* genes both possess a conserved Homeodomain and a Constant domain, NCBI Blast+ [74] was used to identify predicted transcripts encoding these genes in the Trinity assembly by querying for the homeodomains identified in the reference genome. Transcripts were then manually curated by aligning the original RNAseq data to the predicted transcripts using BWA [75] and Hisat2 [76], and visualizing the aligned reads in Geneious to curate a biologically plausible pair of alleles for each gene in each isolate (i.e. binning polymorphisms into two alleles based on agreement with paired-end reads. Later, additional samples with unidentified alleles were also passed through this process until no further alleles could be identified.

The *Pst-P/R* complex is represented by the genes FUN_000740 (STE3.2.1), FUN_005623 (STE3.2.2), and FUN_017677 (STE3.2.3). CDS for these genes was extracted and curated with the RNAseq data from isolate W034, to ensure introns had been properly identified. The resulting CDS were carried forward to allele detection in the same manner as the *Pst-b-HD* locus.

With mating type alleles identified, the alleles of unknown samples were identified using Sourmash [77] to search for representation of mating type allele-derived *k*-mers within the raw nucleotide data for that sample. Sourmash sketch was used to create *k*-mer indexes for curated alleles and for nucleotide sequencing data, using the parameters k=21 and 10X downsampling. Sourmash containment with a threshold of 0 was then used to evaluate each sequence dataset for whether or not it carried each allele, the collective outputs are summarized in Additional File 3: Figure S1 by visualization using the ggplot2 package in R [78] to generate a heatmap with geom_tile from the raw % of *k*-mer matches.

Samples with over 80% *k*-mer identity for a given allele were generally considered to carry that allele. Unusual samples were manually evaluated for a good match between RNA data and curated mating types by aligning RNA to the curated mating type alleles and evaluating the closest match, and in some cases this either prompted curation of another allele, or the allele was left undetermined.

### Clade identification and phylogenetic analysis

Thirteen Canadian samples were previously sequenced by the senior author and reported in Brar et al. [7]; the sequence data was utilized in this project. A further 329 global samples were obtained from other previously published studies [36–38, 40–42, 48–72], in addition to the 44 samples sequenced in this study. Sample origin, mating type, clade, and other metrics are described in Additional File 1.

Illumina reads were aligned to the Pst-130.v2 reference genome using Hisat2 with a minimum score function of L,0,-0.6 and other parameters left default. Alignments were sorted and converted to BAM format using samtools view, and samtools sort [79].

At this stage, quality control was performed on samples by investigating their alignment to the *P. striiformis* f. sp. *tritici* reference genome and to annotated genes within the genome, as well as using a Kraken2 database [80] built from the following preset libraries:

- Fungi
- Plant
- Bacteria

As well as custom libraries constructed from the following genome assemblies:

- GCA_001191645.1*P. triticina*
- GCA_000149925.1*P. graminis*
- GCA_001013415.1*P. arachidis*
- GCA_019395275.1*P. brachypodii*
- GCA_002873125.1*P. coronata*
- GCA_002873275.1*P. coronata*
- GCA_008520325.1*P. graminis*
- GCA_007896445.1*P. hordei*
- GCA_001624995.1*P. horiana*
- GCA_004348175.1*P. novopanici*
- GCA_001263375.1*P. sorghi*
- GCA_002920205.1*P. striiformis*
- GCA_019358815.1*P. triticina*

Of the 17,881 annotated genes in the *P. striiformis* reference genome: average read coverage of >3 reads/bp in fewer than 10,000 genes, low (<20%) *Pucciniales* sample identity in the Kraken2 output, or high (>5%) sample identity from another fungal species in the Kraken2 output were grounds for sample exclusion. Sixteen samples were excluded this way, described in Additional File 1.

For the remaining samples, sorted .bam files were merged into a single mpileup and SNPs called using BCFtools call -m. After calling, SNPs were filtered to intragenic positions using BCFtools filter -R and the set of positions described as exons in the Pst-130.v2 reference GFF3. Following this, informative SNPs were identified using BCFtools filter -i and the parameters:

‘type==’snp’ && AN >600 && AC/AN>0.01 && AC/AN<0.99 && QUAL>20’, which selected for SNPs in positions where at least 300 isolates had sufficient coverage for a call, overall SNP confidence was >20 (*p*=0.05), and the minor allele frequency exceeded 0.01. The remaining 206,376 SNPs were converted to phylip format using the scripts at https://github.com/edgardomortiz/vcf2phylip, then used to generate a maximum-likelihood tree in RAxML [81] using the following settings:

- Mode: -f a
- Model: ASC_GTRGAMMA
- Bootstraps: 1000

The final tree was visualized with IToL and annotated with clades and mating type information using iToL and Adobe Illustrator.

Comparisons of gDNA and RNAseq dataset heterozygosity were performed using R. The vcf file containing all SNPs used in phylogenetic tree construction was queried for just genotype calls and imported into R using read.table. The ratio of heterozygous (0/1) to all alternate allele calls was compared for each sample type, and the mean and standard deviation of this ratio was calculated for each type of data using the default mean and s.d functions.

Comparisons of tree topology were performed using ETE3 compare, in unrooted mode [82].

### Population STRUCTURE analyses

To delineate the clades described in Fig. 2, STRUCTURE analyses were performed on the same data. In brief, SNP information was converted from VCF to strct.in format using PLINK [83] and samples were assigned to presumptive clades by cross-referencing for the closest match in Radhakrishnan et al. [39]. STRUCTURE analyses were run using 2000 MCM and burnin reps, and the assumptions of free admixturing and no association between linked markers for values of *K* between two and 15. The best estimates of *K* were obtained by comparing the change in ln(Pr|X) between values of *K*, as well as by investigating the biological plausibility of the resulting groups. In all cases, the point at which increased values of *K* failed to place any samples into an additional population group agreed with a plateau in ln(Pr|X). After global STRUCTURE comparison was unable to resolve some groups previously identified in other work [28], we performed sub-analyses using related groups identified by the global STRUCTURE analysis. Sub-analyses were able to resolve the missing groups (Fig. 3, Additional File 3: Figure S2).

### Supplementary Information

**Additional file 1.** Datasets generated or used in this study. List of datasets generated or used in this study organised by sample name.  Including year of sampling, country of origin, NCBI SRR, assigned clade, and assigned mating types. 

**Additional file 2. **Nucleotide CDS sequence of HD alleles and STE3 genes of *Puccinia striiformis* f. sp. *tritici* identified in this study, in fasta format.

**Additional file 3: Figure S1. **Presence or absence of mating type alleles across all datasets, evaluated by k-mer match percentage.** Figure S2.  **STRUCTURE analysis of the global *Pst* population, as well as previously listed population subgroups for each tested value of K, as well as ln(Pr|X).

**Additional file 4: **Phylogenetic tree displayed in Fig. 2, in Newick format. Tree file includes branch lengths, bootstraps, and isolate names, but not clade information or mating type annotations. The tree is not rooted in any specific outgroup, and was re-rooted around Psh_93TX-2 in Fig. 2.

**Additional file 5. **Phylogenetic tree generated using only RNAseq datasets, otherwise using the same parameters as in Fig. 2. Tree file includes branch lengths, bootstraps, and isolate names, but not clade information or mating type annotations. The tree is not rooted in any specific outgroup.

## Data Availability

Sequence data generated in this study and Brar et al. [7] was uploaded to the NCBI Short Read Archive under bioproject number PRJNA950118 [84]. The curated coding sequences of *Pst* mating type genes are available as supplementary data to this article.

## Abbreviations

*Pst*: *Puccinia striiformis* f.sp. *tritici*
*PstPr*: *Puccinia striiformis* f.sp. *tritici, Probable Recombinant genetic group*
AA: Amino acid
DNA: Deoxyribonucleic acid
RNA: Ribonucleic acid
CDS: Coding sequence
SNP: Single-nucleotide polymorphism
SP: Single pustule
