# Supplementary material for: Uncovering the history of recombination and population structure in western Canadian stripe rust populations through mating type alleles
Source: BMC Biol. 2023 Oct 25;21:233. doi: 10.1186/s12915-023-01717-9 (PMC10601111; doi:10.1186/s12915-023-01717-9)
Supplement: Supplementary file 3 — Additional file 3: Figure S1. Presence or absence of mating type alleles across all datasets, evaluated by k-mer match percentage. Figure S2. STRUCTURE analysis of the global Pst population, as well as previously listed population subgroups for each tested value of K, as well as ln(Pr|X). [file 12915_2023_1717_MOESM3_ESM.docx]

**Figure S1: Presence or absence of mating type alleles across all datasets, evaluated by k-mer match percentage.** The percentage of unique k-mers (k=21) from each allele contained in each dataset is rendered as a heatmap, with samples showing >80% presence visualised in light blue, samples with >70% presence in green, and samples with lower k-mer containment in brown trending to black at <50% presence. Sample names are given on the left, and allele names at the bottom. *Pst-b-HD* genes are shown as two groups in the left, and matching alleles are in the same order such that the two genes can be easily compared. *STE3* genes are shown separately on the right, and *STE3.2-1* is only identified in genomic datasets.


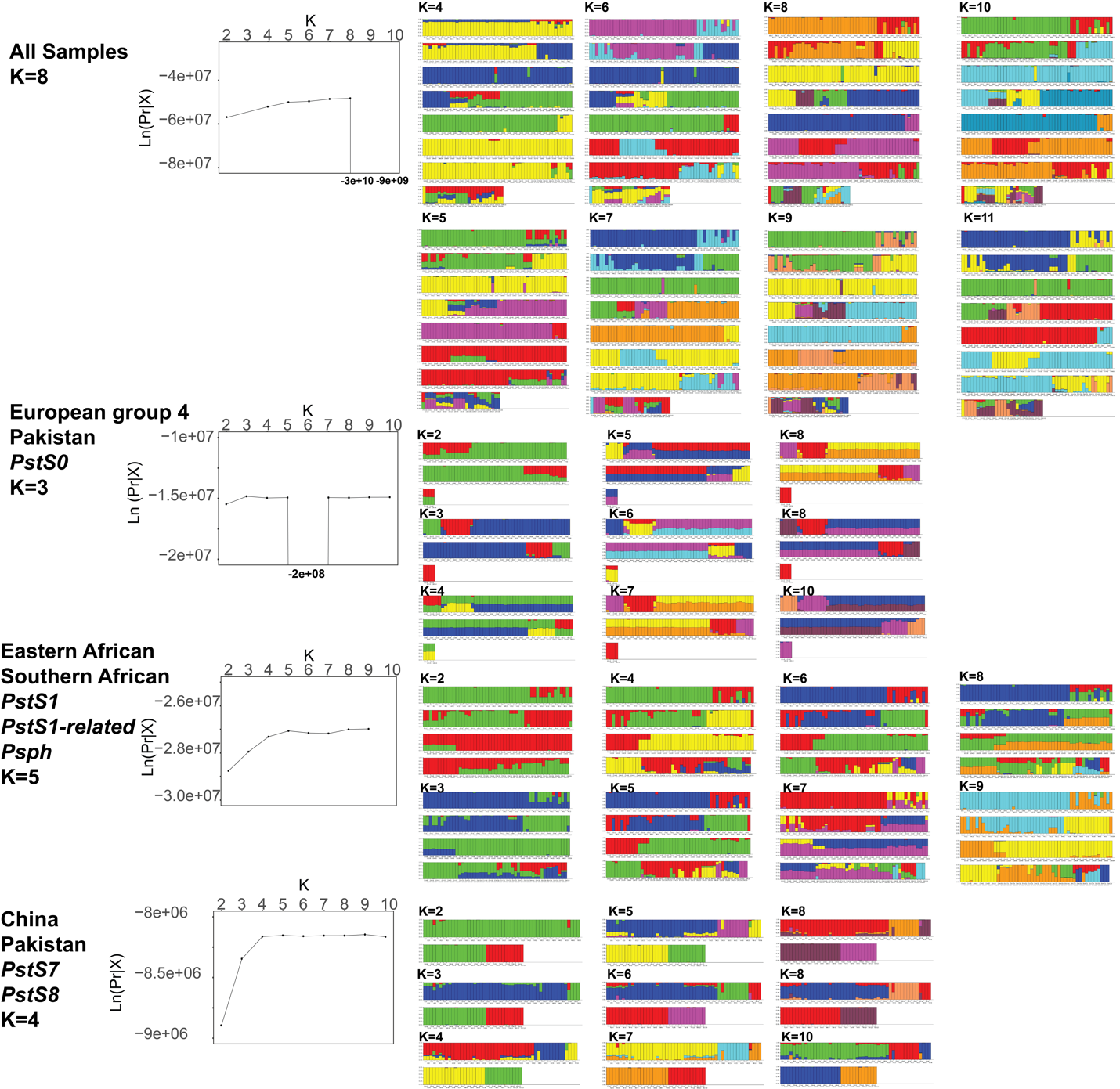


**Supplementary Figure S2. STRUCTURE analysis of the global *Pst* population, as well as previously listed population subgroups for each tested value of K, as well as ln(Pr|X)**. In this case samples are sorted by ID to enable simple comparison between results for different values of K. Where ln(Pr|X) was radically different from the overall trend, the graph has been supplemented with the value.
